# Plant Materials are Sustainable Substrates Supporting New Technologies of Plant-Only-Based Culture Media for *in vitro* Culturing of the Plant Microbiota

**DOI:** 10.1264/jsme2.ME17135

**Published:** 2018-03-29

**Authors:** Elhussein F Mourad, Mohamed S Sarhan, Hassan-Sibroe A Daanaa, Mennatullah Abdou, Ahmed T Morsi, Mohamed R Abdelfadeel, Hend Elsawey, Rahma Nemr, Mahmoud El-Tahan, Mervat A Hamza, Mohamed Abbas, Hanan H Youssef, Abdelhadi A Abdelhadi, Wafaa M Amer, Mohamed Fayez, Silke Ruppel, Nabil A Hegazi

**Affiliations:** 1 Department of Microbiology, Faculty of Agriculture, Cairo University Giza 12613 Egypt; 2 Regional Center for Food & Feed (RCFF), Agricultural Research Center Giza 12619 Egypt; 3 Department of Microbiology, Faculty of Agriculture & Natural Resources, Aswan University Aswan 81528 Egypt; 4 Department of Genetics, Faculty of Agriculture, Cairo University Giza 12613 Egypt; 5 Department of Botany and Microbiology, Faculty of Science, Cairo University Giza 12613 Egypt; 6 Leibniz Institute of Vegetable and Ornamental Crops (IGZ) Grossbeeren 14979 Germany

**Keywords:** plant-only-based culture media, plant microbiome/microbiota, wild desert barley, microbial biomass, rhizobacteria culturability

## Abstract

In order to improve the culturability and biomass production of rhizobacteria, we previously introduced plant-only-based culture media. We herein attempted to widen the scope of plant materials suitable for the preparation of plant-only-based culture media. We chemically analyzed the refuse of turfgrass, cactus, and clover. They were sufficiently rich to support good *in vitro* growth by rhizobacteria isolates representing *Proteobacteria* and *Firmicutes*. They were also adequate and efficient to produce a cell biomass in liquid batch cultures. These culture media were as sufficient as artificial culture media for the cultivation and recovery of the *in situ* rhizobacteria of barley (*Hordeum murinum* L.). Based on culture-dependent (CFU plate counting) and culture-independent analyses (qPCR), mowed turfgrass, in particular, supported the highest culturable population of barley endophytes, representing >16% of the total bacterial number quantified with qPCR. This accurately reflected the endophytic community composition, in terms of diversity indices (S′, H′, and D′) based on PCR-DGGE, and clustered the plant culture media together with the qPCR root populations away from the artificial culture media. Despite the promiscuous nature of the plant materials tested to culture the plant microbiome, our results indicated that plant materials of a homologous nature to the tested host plant, at least at the family level, and/or of the same environment were more likely to be selected. Plant-only-based culture media require further refinements in order to provide selectivity for the *in vitro* growth of members of the plant microbiome, particularly difficult-to-culture bacteria. This will provide insights into their hidden roles in the environment and support future culturomic studies.

The consortia of microorganisms, the microbiota, and their collective genomes, the microbiome, reside in and on all multicellular eukaryotes, and they engage in the host’s nutrition and health through their rich diversity and multiple interactions (27, 29). In plants, previous studies reported significant differences in the relative abundance of the community compositions of leaves and stems (the phyllosphere) and roots (the rhizosphere). Characterizations have also been made according to residential locations; ectophytes reside outside the plant, while endophytes reside within it (20). Metabolite enrichment by roots in the form of exudates, slough offs, and depositions in the rhizosphere necessitates the recruitment of sufficiently compatible free soil microbes towards a nutrient-rich rhizosphere. This effect is prominent in plants that naturally grow under stress environments, particularly deserts (10, 34, 35). This interaction between the rhizospheric milieu and host plant may be summarized as a harmonious bidirectional relationship whereby the latter poses as a suitable niche that characterizes its community through its genotype and metabolic secretions, while the former markedly enhances the host’s nutrition and health through multiple functions, *e.g.* hormone production, nitrogen fixation, nutrient availability and uptake, and the antagonism of pathogens (29). These multi-functional groups of the plant microbiome, particularly nitrogen-fixers and plant growth-promoting rhizobacteria (PGPR), are currently being investigated and development programs are being conducted in order to maximize their contribution to agricultural sustainability and good agricultural practices (GAP) (1, 22, 51). Therefore, the sufficient biomass production of rhizobacteria is a prerequisite for the large-scale manufacture of bio-preparates, *e.g.* biofertilizers and biocontrol agents. This is of importance for addressing crucial economic issues regarding the development of agro-resources and agricultural productivity, particularly in developing countries.

Advances in molecular biology techniques with the development of culture-independent techniques have highlighted the joint genomic, transcriptomic, and proteomic interactions that influence the culturability of microorganisms (47). Despite the high-throughput nature of these culture-independent approaches for community profiling (42), previous studies demonstrated their inherent bias that results in a superficial view of microbial community compositions (24). Unculturable populations account for more than 90% of a given ecosystem, and represent diverse groups of microbes with overlooked functional niches. Previous studies attempted to cultivate these unculturable populations in order to obtain a better understanding of their roles in biogeochemical cycles, soil fertilization, and plant-microbe interactions (40). The existence of unculturable bacteria has emphasized the need to develop approaches that replicate the conditions of a complex ecosystem that suitably accommodates a wide spectrum of bacterial populations *in vitro* (9,13). Nutritional compositions in the form of complex matrices have been recognized to orchestrate the diversity of microbes in a given ecosystem, either terrestrial or aquatic (11, 50). Consequently, it is imperative for microbiologists to tailor an artificial medium for *in vitro* study and/or grow a given bacterial isolate or community (5). This satisfies the nutrients necessary for bacterial growth, such as carbon, nitrogen, and phosphorus sources, in addition but not limited to macro- and micro-elements at varying concentrations that may include other additives depending on strain specificity. The continuous pursuit and development of cheap, sustainable, and natural sources such as agro-industrial byproducts (including effluents, whey, molasses, and bagasse) or plant substrates and remains (such as hay, straw, and sawdust) for *in vitro* growth and biomass or essential metabolite production is of substantial economic and environmental relevance. This is not only for financially-limited labs and industries, but also as an efficient and convenient scheme towards the recycling process and resource sustainability (6). The use of suitable plant materials for the *in vitro* growth and biomass production of rhizobacteria, in the form of plant-only-based culture media, represents a novel approach in culturing technologies for examining the plant microbiome. Based on various plant materials, these culture media have proven to be ample and sustainable sources of nutrients (18, 32, 41, 43, 52). Further advances in this respect will contribute to the discovery of new members of rhizobacteria including promising biomass production needed for biotechnological applications in support of plant productivity and environment sustainability.

The present study amplifies the spectrum of our previous findings on the sole use of plant-only-based culture media to support the *in situ* recovery and *in vitro* growth as well as biomass production of representative isolates of rhizobacteria (18, 32, 41, 43, 52). The plant materials tested are refuse of mowed turfgrass, cactus pads, and Egyptian berseem clover plants. Since they are cheap, available, and natural sources of nutrients, we investigated their potential for the *in vitro* culturing of rhizobacteria and production of value-added biomass. Furthermore, the common and endogenous wild desert barley (*Hordeum murinum* L.) was selected as a host plant to investigate the suitability of the tested plant-only-based culture media for studying the *in situ* community composition diversity of rhizobacteria in various root compartments. In order to achieve this, we applied culture-dependent and -independent approaches, namely CFU counts, Polymerase Chain Reaction-Denaturing Gradient Gel Electrophoresis (PCR-DGGE) of the 16S rRNA gene, and quantitative real-time PCR (qPCR).

## Materials and Methods

### The tested host plant

The tested host plant was the wild desert barley (*Hordeum murinum* L.; Family *Poaceae*) naturally grown in the deserts of Borg El-Arab, 45 km southwest of Alexandria, Egypt (N30°57,849 E29°40,140). Samples representing the whole root system of fully grown plants were collected together with the adjacent soil. Free soil in between plants was also sampled. Microbiological analyses were conducted within 18 h of sample collection.

### Culture media

#### Plant-only-based culture media

The following plant refuse were tested: mowed blades of turfgrass (*Paspalum vaginatum* Sw.; Family *Poaceae*) obtained from the Shooting Club, El-Dokki, Giza, Egypt; discarded mature stem pads of cactus prickly pears (*Opuntia ficus-indica* L. Mill.; Family *Cactaceae*) from El-Orman Botanical Garden, Giza, Egypt; leftovers of the third cutting of fully grown Egyptian berseem clover (*Trifolium alexandrinum* L.; Family *Fabaceae*) from the experimental fields of the Faculty of Agriculture, Cairo University, Giza, Egypt. Plant materials were prepared in the form of slurry homogenates and/or dehydrated plant powder for the formulation of plant-only-based culture media. In order to compare the nutritional profiles of the dehydrated plant powders tested, chemical analyses were performed by the certified Regional Center for Food and Feed (RCFF), Agricultural Research Center (ARC), Giza, Egypt, (rcff. com.eg/ISO%20Accriditation/Scope.htm). Analyses included total crude protein, total crude fiber, total ash, total carbohydrates, amino acids, vitamins, and minerals (macro- and micro-nutrients).

#### Slurry homogenates of tested plants

Fresh parts of leftover clover plants and cactus pads were sliced and blended in a Waring blender with minimum amounts of distilled water to facilitate their homogenization; *ca.* 10:1 plant: water (w/v) for clover, and without distilled water in the case of cactus pads. The resulting slurry was stored at −20°C until used. Upon use, slurry homogenates were diluted (homogenate: distilled water, 1:20 [v/v]) and then used without any amendments for the preparation of plant-only-based culture media.

#### Dehydrated plant powders

Mowed turfgrass, leftover clover plants, and discarded cactus pads were dehydrated in sunlight for 2–4 d, and then subjected to oven drying at 70°C for 24–48 h. Dehydrated plant materials were mechanically grinded to pass through a 2.0-mm sieve in order to obtain fine dehydrated powder. Aliquots of 1, 2, 4, or 8 g of dehydrated powders were soaked in 1 L of lukewarm distilled water to obtain liquid plant infusions, which were then autoclaved for further extraction and sterilization.

The freshly prepared plant slurry homogenates and dehydrated powders described above were used to prepare the tested plant-onlybased culture media in their liquid forms. Regarding solid culture media, agar-agar was added (2% [w/v]). Liquid and solid culture media were pH adjusted to 7.0, then autoclaved at 121°C for 20 min.

#### Artificial culture media

Rich nutrient agar (21) and soil extract agar (36), as well as N-deficient combined carbon-source medium (CCM) (17) were used throughout this study.

#### Nutrient agar

Nutrient agar contained the following (g L^−1^): Beef extract, 3.0; peptone, 5.0; glucose, 1.0; yeast extract, 0.5; agar, 15; pH, 7.2.

#### Soil extract agar

Soil extract agar contained the following (g L^−1^): Glucose, 1.0; peptone, 1.0; yeast extract, 1.0; K_2_HPO_4_, 1.0; soil extract, 400 mL; tap water, up to 1 L; agar, 15; pH 7.2. Fresh soil extract was prepared according to Parkinson *et al.* (36); 400 g of fertile soil was added to 1.0 L of tap water, followed by autoclaving at 121°C for 60 min. Upon cooling to room temperature, the supernatant solution was decanted and filtered through Whatman filter paper and its volume was brought to 1.0 L using distilled water.

#### N-deficient CCM

N-deficient CCM comprised the following (g L^−1^): Glucose, 2.0; malic acid, 2.0; mannitol, 2.0; sucrose, 1.0; K_2_HPO_4_, 0.4; KH_2_PO_4_, 0.6; MgSO_4_, 0.2; NaCl, 0.1; MnSO_4_, 0.01; yeast extract, 0.2; fermentol (a byproduct of corn-steep liquor), 0.2; KOH, 1.5; CaCl_2_, 0.02; FeCl_3_, 0.015; Na_2_ MoO_4_, 0.002. In addition, CuSO_4_, 0.08 mg; ZnSO_4_, 0.25 mg; in addition, 0.6 mL of sodium lactate (50% v/v), was added per liter. Finally, agar (15 g L^−1^) was added and pH adjusted to 7.2.

#### In vitro growth of pure isolates of rhizobacteria on plant-only-based agar culture media

Sixteen representative isolates of the rhizobacteria families, *Bacillaceae*, *Enterobacteriaceae*, and *Pseudomonadaceae*, were obtained from the culture collection of the Department of Microbiology, Faculty of Agriculture, Cairo University, Giza, Egypt (Table S1). Isolates were initially inoculated into semi-solid CCM test tubes, and the resulting 2–4-d-old cultures were microscopically examined for growth and purity. Aliquots of 50 μL of the bacterial cultures were spread on the surfaces of agar plates representing all of the tested plant-only-based agar plates and nutrient agar. Plates were incubated at 30°C for 7 d, and the visual growth index recorded was as follows: 0, no growth; 1, scant (discontinued bacterial lawn, with scattered colonies); 2–3, good (continued bacterial lawn), and 4–5, very good (continued and very dense bacterial lawn).

#### Biomass production of pure isolates of rhizobacteria in batch cultures prepared from liquid plant-only-based culture media

PGPR isolates representing three major genera: *Klebsiella oxytoca*, *Enterobacter agglomerans*, and *Azospirillum brasilense*, were selected. Their growth was monitored in liquid plant-only-based batch culture media using suitable plant dehydrated powders (4 g L^−1^) and/or slurry homogenates (diluted with distilled water 1:20 [v/v]) of the tested plant refuse. Artificial liquid CCM was included for comparison. Liquid culture media were prepared (100 mL in 250-mL Erlenmeyer flasks) in 3 replicates for each treatment, inoculated with each of the tested isolates (2% [v/v]), and incubated at 30°C in a rotary shaker (100 rpm) for up to 7 d. Periodic samples from the resulting batch cultures were surface-inoculated on CCM agar plates, in triplicate, for CFU counts. Growth rates (Eq. 1) and doubling times (Eq. 2) were calculated (38):

Eq. 1 $$\text{Growth rate }(K)=\text{log}N_{t}-\text{log}N_{o}/\text{log}2(T_{t}-T_{o})$$

Eq. 2 Doubling time (dt)=1/K,

Where *N_o_*=viable cell counts at *T_o_*, *T_o_*=time at the beginning of the assessment, *N_t_*=viable cell counts at Tt, Tt=time after (t) h

Computation of the doubling time was performed according to Roth V. 2006 Doubling Time Computing: http://www.doubling-time.com/compute.php

#### Culture-independent estimation of bacterial copy numbers of root endophytes by qPCR

After the surface sterilization of barley roots (43, 52), *ca.* 5 g of roots was soaked in sterilized bottles containing 45 mL of the basal salts of CCM medium as a diluent, and were referred to as mother cultures. Aliquots of 1.5 mL, in 4 replicates, of this original root suspension were centrifuged at 10,000 rpm for 10 min. According to Sarhan *et al.* (43), DNA was isolated from the roots, diluted (1:10 [v/v]), and analyzed in duplicate. The amplification and detection of DNA by real-time PCR were performed with SYBR Green™ technology and measured in the CFX96 Touch™ Real-Time PCR Detection System (Bio-Rad, Hercules, CA, USA) using optical grade 96-well plates (43). The universal bacterial degenerate primers 519f (CAGCMGCCGCGGTAANWC) and reverse 907r (CCGTCAATTCMTTTRAGTT) were used to enumerate copy numbers (26, 46). The PCR reaction mixture contained 12.5 μL SYBR^®^ green master mix (Bio-Rad), 2 μL DNA (*ca.* 3–15 ng), 2.5 μL of 3.3 pmol of each primer, and 5.5 μL PCR water in a final volume of 25 μL. The thermal amplification cycling program was initiated by a 3-min initial denaturation at 95°C, then 40 thermal cycles of: denaturation at 95°C for 15 s, annealing at 53°C for 30 s, and extension at 72°C for 42 s; followed by melting curve construction by increasing the temperature from 53°C to 95°C with fluorescence detection for every 0.5 K. A 6-point standard curve starting from 2.5E+2 to 2.5E+7 was constructed using a 407-bp purified amplicon of the *E. coli* 16S rRNA gene. Bacterial cell numbers were obtained indirectly assuming 3.6 as the average number of rRNA operons per cell (23, 44). The mean value of qPCR cell numbers indirectly obtained for 4 replicates was log 9.37±0.001 g^−1^ root dry weight. Culturability (C) was calculated as a percentage (%) by relating the total number of CFUs (N) developed on agar plates to the total bacterial numbers measured by qPCR (T):

$$\text{C }(\%)=\frac{N}{T}*100$$

#### Culture-dependent quantification of in situ rhizobacteria associated with barley roots

Microbiological analyses included samples of the ecto-rhizosphere representing the root surfaces together with closely adhering soil (35). Furthermore, carefully-washed and surface-sterilized roots were used to prepare endo-rhizosphere samples (43, 52). Free soil was also included for comparison. The first dilutions of free soil as well as the ecto- and endo-rhizosphere were prepared by adding 5 g of the respective samples to 45 mL of the sterilized diluent (the basal salts of CCM culture medium). Further serial dilutions of all tested spheres were prepared, and aliquots (200 μL) of suitable dilutions were surface inoculated on agar plates, with 3 replicates, prepared from all tested culture media. The plant-only-based culture media tested were those prepared from the crude slurry homogenates (diluted with distilled water, 1:20 [v/v]) of clover and cactus as well as the dehydrated plant powders of clover, cactus, and turfgrass (4 g L^−1^). The artificial culture media of nutrient agar, soil extract, and CCM were included for comparison. Plates were incubated at 30°C for 2–4 d. The suspended roots and free soil were oven-dried at 70°C and 105°C, respectively, to obtain their dry weights for total CFU calculations.

### DGGE analysis for comparing culture-dependent and culture-independent rhizobacteria populations

#### DNA extraction from CFU harvests and root suspensions

All CFUs developed on agar plates (>30–300 CFUs plate^−1^), in 3 replicates, representing various culture media were harvested using 7 mL of 0.05 M NaCl solution. The CFU harvest as well as previously prepared original root suspensions were centrifuged at 13,000 rpm for 10 min. DNA was extracted from the resulting pellets using the i-genomic Soil DNA Extraction Mini Kit (iNtRON Biotechnology, Kyungki-Do, Korea). DNA concentrations were measured using the NanoDrop 2000 UV-Vis Spectrophotometer (Thermo Fisher Scientific, Waltham, MA, USA).

#### Amplification of the 16S rRNA gene and nested PCR for DGGE fingerprinting

The protocols for 16S rRNA gene amplification and nested PCR of the V3-16S rRNA gene (30, 31), adapted by Sarhan *et al.* (43), were used. 16S rRNA gene amplification from DNA extracted, either from the CFU harvest or original root suspensions, was performed with the Bio-Rad C1000 Touch™ Thermal Cycler (Bio-Rad, Hercules, CA, USA).

DGGE was performed using the Bio-Rad Dcode Mutation Detection System (Bio-Rad). Aliquots of 10 μL of each sample were mixed with 3 μL of a 6×loading dye (glycerine, xylene cyanol, bromophenol blue), then heated at 95°C for 5 min, and stored at 65°C until loading. Amplicons were electrophoresed on an 8% acrylamide gel containing a 30 to 70% denaturing gradient of formamide and urea with 1×TAE buffer. After 3 min of initial migration at 200 V to push the sample into the gel, DGGE was conducted at 60°C for 20 h at 50 V. The gel was stained for 30 min with the SYBR^®^ Gold Nucleic Acid Gel Stain (Life Technologies, Darmstadt, Germany), photographed, and analyzed for DGGE band profiles with the UV gel documentation system (Biometra GmbH, Göttingen, Germany). A self-created standard was constructed according to Sarhan *et al.* (43) and included in every DGGE run.

### Statistical analyses

Graphical representations were performed using GraphPad Prism 7.0 (GraphPad Software, La Jolla, CA, USA). An F-test factorial ANOVA was performed using STATISTICA V10 (Statsoft, Tulsa, OK, USA) to analyze the results obtained for CFU counts. DGGE fingerprints were analyzed using Phoretix 1D pro software (TotalLab, Newcastle upon Tyne, UK). A principal co-ordinates analysis (PCoA) was conducted using GenALEx 6.5 (MS Excel 2016 ADD-IN) (37). The diversity indices, Richness (S′), Shannon (H′), and Simpson (D′) indices, were calculated using PAST software v 3.0 (16). A heteroscedastic Student’s *t*-test was performed to compare the DGGE distances of plant-only-based culture media vs. barley roots and artificial culture media vs. barley roots. Deviations in the alpha diversity indices of all tested culture media from barley roots were calculated as a measure of the relatedness of culturable populations to native communities of barley roots.

## Results and Discussion

### The rich and diverse nutritional matrix of tested plant materials used for the preparation of plant-only-based culture media

**Fig. 1:**
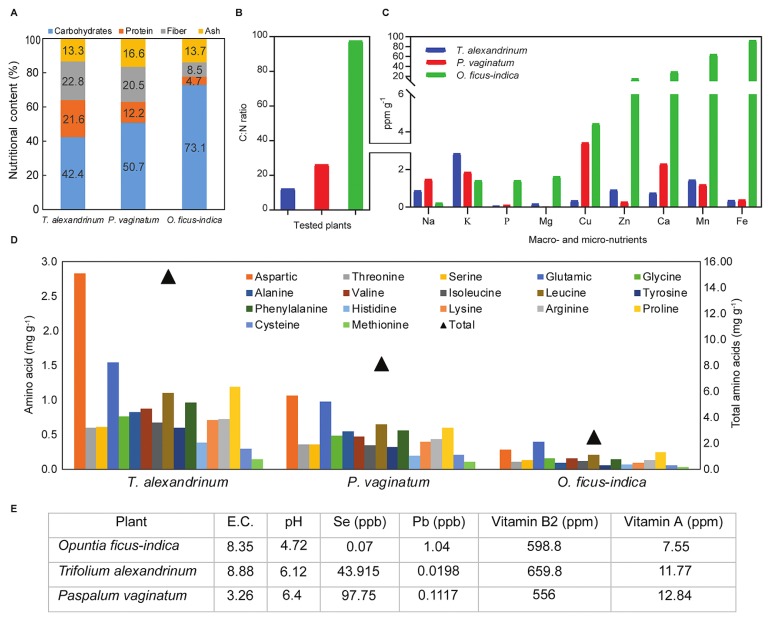
Nutritional profiles of tested plant refuse based on multiple chemical analyses: **A**, Major contents of carbohydrates, proteins, fiber, and ash; **B**, C/N ratio; **C**, contents of macro- and micro-nutrients (ppm g^−1^ dehydrated powder); **D**, total and individual amino acids contents (mg g^−1^ dehydrated powder); **E**, remaining measured chemical parameters of dehydrated powders prepared for tested plants (*Trifolium alexandrinum*, *Paspalum vaginatum*, and *Opuntia ficus-indica*) and used for the preparation of plant-only-based culture media.

The varying nutritional profiles of the three tested plant materials used in the preparation of plant-only-based culture media are shown in Fig. 1. They were rich in multiple nutrients required for the growth of tested rhizobacteria, *e.g.* carbon and nitrogen sources, macro- and micro-elements, amino acids, and vitamins. They also represented and mimicked the varying natural and diverse nutritional matrix of the plant roots of various genotypes (*Fabaceae*, *Poaceae*, and *Cactaceae*) grown in different ecosystems (semi-arid deserts and Nile Delta). The C/N ratio ranged between 12.2 and 97.6, and was the narrowest for clover and widest for cactus. Analyses of the tested mowed turfgrass (Fig. 1) were similar to those reported in the literature, having the following compositions: C/N ratio, 26.2; protein content, 11.4%; fiber, 19.2%; ash, 15.6% (28). The corresponding contents of cactus pads were 97.6, 4.7%, 8.5%, and 13.7%, which were similar to those reported by Hernández-Urbiola *et al.* (19). With reference to its desert habitat, these cactus pads were particularly rich in macro- and micro-nutrients.

### Very good *in vitro* growth of rhizobacteria on tested plantonly-based agar culture media

**Fig. 2:**
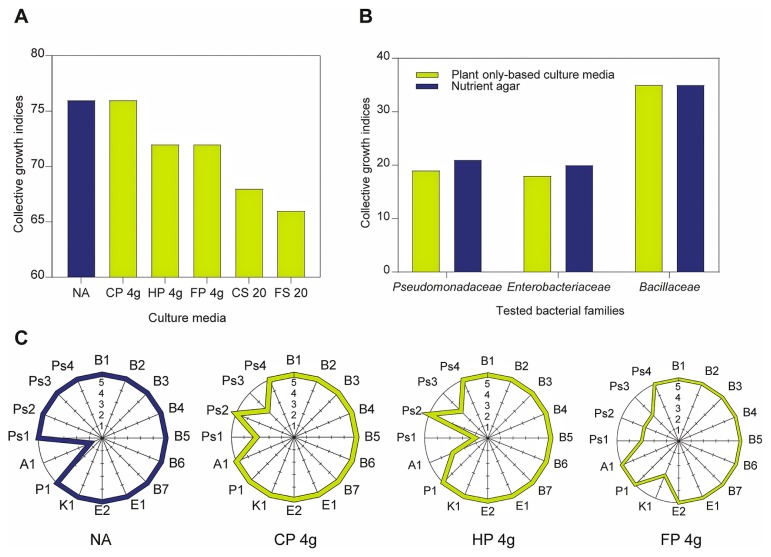
Growth of rhizobacteria isolates on agar plates of plant-only-based culture media prepared from plant slurry homogenates and powders as well as nutrient agar: **A**, Collective growth indices of tested rhizobacteria isolates, with each depicted value representing the sum of growth indices measured for all 16 isolates; **B**, varying growth behavior in terms of collective growth indices of all tested rhizobacteria families; **C**, radar presentation of all tested rhizobacteria isolates on plant-only-based culture media (**CP**, clover powder; **HP**, turfgrass powder; **FP**, cactus powder; **CS**, clover slurry; **FS**, cactus slurry) and nutrient agar (**NA**). Tested isolates: **B1**, *B. circulans* B43; **B2**, *B. circulans* 3B; **B3**, *B. licheniformis* En17/3; **B4**, *B. macerans* 21B; **B5**, *B. Polymyxa* 1E; **B6**, *B. polymyxa* 30B; **B7**, *B. subtilis* NA20; **E1**, *E. agglomerans* K4; **E2**, *E. agglomerans* K3; **K1**, *Klebsiella* sp. 31Sh; **P1**, *Pantoea* sp. En5/1; **A1**, *Azotobacter chroococcum* B4; **Ps1**, *P. aeruginosa* Arbo17; **Ps2**, *P. cepacia* 34Sh; **Ps3**, *P. fluorescens* Arbo4; **Ps4**, *Pseudomonas* sp. B6.

The sixteen tested rhizobacteria isolates exhibited good to very good growth on the agar plates of tested plant-onlybased culture media prepared from all three plant materials. Growth indices were similar to those reported for standard nutrient agar (Fig. 2A). Slower growth was reported with plant slurry preparations, and may be attributed to their concentrated nutritional contents creating osmotic stress environments (8, 24, 39).

In order to evaluate the growth preferences of rhizobacteria at the family level, the cumulative growth indices of all tested isolates were considered on each of the tested plant-onlybased culture media. The results obtained showed a particular preference towards the growth of *Bacillaceae* (*Firmicutes*), followed by *Enterobacteriaceae* and *Pseudomonadaceae* (*Proteobacteria*) (Fig. 2B). These results extend the findings of Youssef *et al.* (52) who clearly demonstrated that plantonly-based culture media supported better growth within the phyla *Firmicutes*, *Proteobacteria*, and *Bacteroidetes*. The promiscuous nature of the plant refuse tested to support a wide array of rhizobacteria encourages their use for the purpose of culturing as well as biomass production for the formulation of bio-preparates, *e.g.* biofertilizers and bio-pesticides (1, 3, 4).

Among the 20 different combinations used for the preparation of plant-only-based culture media, *viz.* dilutions of 1:10 up to 1:40 of slurry homogenates and weights of 1 to 8 g L^−1^ of dehydrated plant powders (Fig. S1), the three most superlative culture media were the use of 4 g L^−1^ powder of clover, cactus, and turfgrass (Fig. 2C). Nutrient agar did not support the growth of *Azotobacter chroococcum*, which was in contrast to the good growth observed on plant-only-based culture media. This was explained by the complex nutritional requirements of nitrogen-fixing *Azotobacter* spp. and *Rhizobium* spp. when grown *in vitro* (7, 33, 45); they require higher contents of a C source, trace elements (Fe, Mo, and Mn), amino acids, and vitamins as growth factors for the efficient function of their *nif* genes. These nutritional requirements were abundant in the plant materials tested, particularly cactus (Fig. 1). Such nutritional requirements were previously confirmed for representative isolates of *Azotobacter chroococcum*, *Azospirillum brasilense*, as well as fast-growing rhizobia that produced sufficient microbial biomass when grown on rich agro-industrial wastes *e.g.* bakers’ yeast effluents (3, 4).

### Copious biomass production of rhizobacteria in batch cultures prepared from liquid plant-only-based culture media

**Fig. 3:**
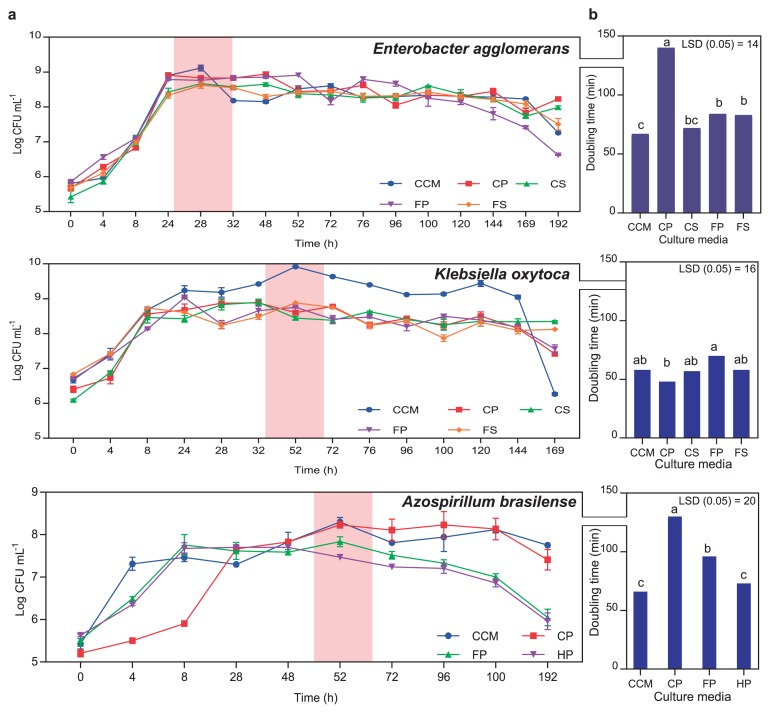
**A**, Growth and cell biomass production of *Klebsiella oxytoca*, *Enterobacter agglomerans*, and *Azospirillum brasilense* in liquid batch cultures prepared from plant only-based culture media based on slurries and/or powders of clover, cactus, and turfgrass compared to the artificial CCM culture medium; **B**, calculated doubling times in min; each figure represents 2 points at the log phase for 2 replicates of batch cultures (*n*=4); significant differences (*P*<0.05) are indicated by different letters. (**CP**, clover powder; **HP**, turfgrass powder; **FP**, cactus powder; **CS**, clover slurry; **FS**, cactus slurry)

When grown in liquid batch cultures, *K. oxytoca* exhibited very good growth in culture media prepared from slurry homogenates and the powders of clover and cactus, with similar growth velocities to that of artificial CCM culture medium (*dt*=48–58 min) (Fig. 3B). Cell survivability in plantonly-based culture media was extended for up to 7 d (Fig. 3A). Regarding *E. agglomerans*, growth was similar on all culture media tested (*dt*=72–84 min), except for clover powder (*dt*=140 min). While doubling times reported for clover slurry were not significantly different from that for the standard CCM culture medium (*dt*=67–72 min), other doubling times were significantly longer for other plant culture media. However, cell survivability was maintained for longer times in all plant culture media tested (Fig. 3A). Although *A. brasilense* displayed normal growth on all plant culture media tested, turfgrass powder supported the best growth at the same magnitude of CCM (*dt*=66–73 min) (Fig. 3B). In general, the plant culture media tested produced a sufficient cell biomass of >10^8^–10^9^ cells mL^−1^ culture as early as day 1 of the incubation of *E. agglomerans*, whereas a slightly longer time of 2–3 d was observed for *K. oxytoca* and *A. brasilense*.

The plant-only-based culture media tested supported normal and good growth velocities as well as cell biomass production that were similar to those of artificial CCM culture medium. However, the rate of growth and efficiency of biomass production are a function of the plant material used and growing bacterium. These results are encouraging from an industrial and economical perspective because the proposed culture media are based on plant materials that are safe, available, and cheap raw materials that adequately house an array of microbial strains and communities, *i.e.* without any additional amendments. Efficient biomass production partly relies on the production time and speed of nutrient dynamics in the growth medium (2), particularly in the case of the rich store in plant-only-based culture media. Therefore, the reported generation times of the tested rhizobacteria isolates grown in plant-based culture media were, in most cases, similar to those of artificial culture media, and are of potential use in biomass production (Fig. 3B). The diversity of carbon and nitrogen sources in plant-based culture media has an impact on the efficiency of multiple and successive metabolic pathways of the organisms tested, which secures continuous and sustained growth over the observed stationary phases. These findings were carefully examined for rhizobacteria, including rhizobia (14). Furthermore, an implication on survivability opens possibilities for strain preservation while simultaneously maintaining cell viability and genetic stability for longer periods of time in a native-culture medium *e.g.* similar to the proposed plant-only-based culture medium (39).

Previous studies successfully demonstrated the suitability of agro-industrial effluents such as baker’s yeast effluent for the biomass production of rhizobacteria (3, 4). Another opportunity may be capitalized on through the application of the plant-only-based culture medium concept or together with other agro-industrial residues (*e.g.* molasses, corn steep liquor, whey, bagasse, and other effluents) in order to improve the technical and economic aspects of biomass production. Further studies are needed in order to establish whether biomass and metabolite production efficiency may be improved by modifying growth conditions such as the pH and temperature of the process (49).

### Excellent recovery of in situ rhizobacteria of barley on plantonly-based culture media, and percentage of culturability related to total bacterial numbers quantified by qPCR

In consideration of the development of desert agriculture in the last two decades, wild desert barley (*Hordeum murinum* L.) was selected as the host plant tested to investigate the community composition of both the outer (ecto-rhizosphere) and inner (endo-rhizosphere) root compartments. This plant is among the rich naturally-grown *Poaceae* flora in the semiarid environments of the western deserts of Egypt. It represents a significant microbial and genetic pool for the improvement of domesticated varieties in respect of resistance to stronger biotic and abiotic stresses (12).

Rhizobacteria that associated with the roots of barley were detected using culture-dependent (*i.e.* CFU count) and culture-independent (qPCR) methods (Table 1). Regarding culture-dependent methods, the plant-only-based culture media tested supported the growth of well-developed and distinct colonies and microcolonies of rhizobacteria associated with barley roots (Fig. S2, S3). However, most of the fast-growing colonies developed on standard nutrient agar and soil extract agar media creeped over and smeared developing microcolonies. In terms of apparent morphology, colonies grown on plant-only-based agar plates were more diverse, limited, and smaller in diameter (Fig. S3). Statistical analyses of total CFU counts indicated significant differences (*P*<0.05) attributed to the single effects of culture media, incubation times, and two-way interactions (culture media × incubation time). Plantonly-based culture media significantly supported the good development of CFUs, similar to artificial culture media.

In free soil, the culturable population of bacteria ranged between 5.5 and 6.8 Log CFU g^−1^, with a significant growth preference on turfgrass powder followed by the remaining plant-based culture media tested as well as artificial culture media. The particular enrichment of rhizobacteria was reported in the ecto-rhizosphere (7.9–9.8 Log CFU g^−1^) and culturable populations were in the descending order of soil extract, followed by culture media based on the powders of cactus, turfgrass, and clover (Table 1). Plant-only-based culture media were generally of no additional advantage for culturing free soil bacteria, being as good as the other artificial culture media tested. This was in contrast to the higher affinity towards culturing the bacterial population of the inner compartment (endo-rhizosphere) of the root. The highest culturable population of endophytic bacteria was reported for plantonly-based culture media prepared from turfgrass powder (8.59 Log CFU g^−1^) followed by desert cactus powder and slurry.

In order to measure the efficiency of culturability at the level of endophytes, amplification of the 16S rRNA gene for barley roots using qPCR was performed. The resulting mean total bacterial cell number was Log 9.37±0.001 g^−1^, representing culturable and unculturable populations (Table 1). In this inner root compartment, the culturable population that developed on turfgrass powder culture medium represented 16.3% of the total bacterial cell number calculated by qPCR. This value was at least twice as high as those of all other culture media tested. This result may be interpreted with regards to the nutritional suitability of turfgrass that contained a number of C and N substrates, macro- and micro-nutrients, and a wide array of growth factors represented by amino acids and vitamins (Fig. 1). This further highlights the unique nutritional profile of *Paspalum vaginatum* as an abundant forage that was reported previously under different environmental conditions (28). Of interest is the higher levels of selenium among other micro-nutrients, which potentially boosts the growth of endophytes either *in vitro* or *in situ*, and indicates a strong plant-microbiome interaction (48). Overall, even with the best culturability percentage reported on turfgrass powder culture media, unculturables still constituted more than 80% of rhizobacteria (Table 1).

With other plant materials, culturability increased slightly within the range of >3.3% to 6.1%. The lowest culturability was reported for artificial culture media (3.4–3.9%). These results not only provide more depth to our previous studies (18, 32, 41, 43, 52), but also offer an insight into the relevance of plant materials selected for the preparation of the plant-only-based culture medium and the respective plant sphere or niche under investigation. All tested plant materials used for culture medium preparation generally supported the good development and culturability of endophytes. However, our results recommend the use of plant materials of a homologous nature, at least at the family level, and/or of the same environment to the tested host plant (33). A justification that may explain the favorability of the culture medium based on mowed turfgrass to support the highest development of culturable endophytic rhizobacteria of barley is that both belong to the family *Poaceae*.

### DGGE analysis indicated the distinguished community composition of endo-rhizobacteria developed on plantonly-based culture media

**Fig. 4:**
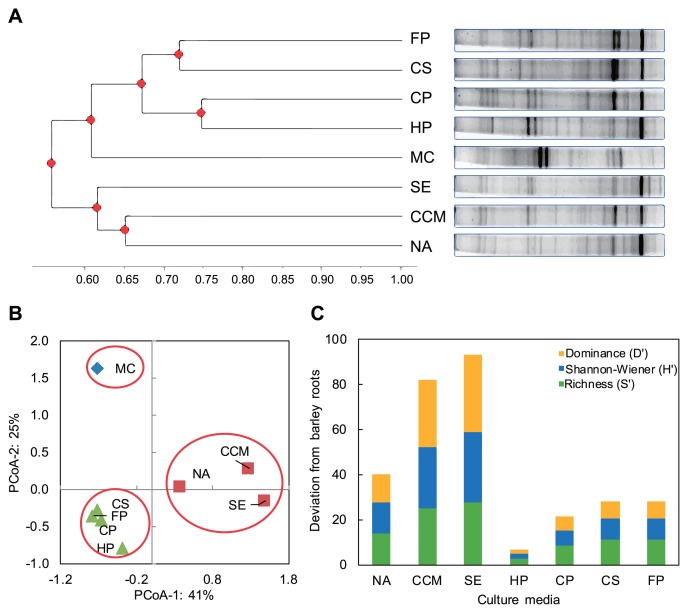
PCR-DGGE cluster analyses (based on Euclidean distance) of rhizobacteria communities associated with barley roots and CFU harvest of different agar culture media plates: **A**, UPGMA cluster analysis; **B**, principal coordinates analysis generated from unweighted DGGE banding data; **C**, Deviations of different culture media calculated for diversity indices as percentages from the total deviation from barley roots. **NA**, nutrient agar; **CCM**, N-deficient combined carbon-source medium; **SE**, soil extract agar; **CS**, clover slurry; **FP**, cactus powder; **CP**, clover powder; **HP**, turfgrass powder; **MC**, mother culture (surface-sterilized barley roots).

The community compositions of the endophytic rhizobacteria of barley roots revealed by culture-independent (qPCR analysis of surface-sterilized roots) and culture-dependent populations (CFUs developed on agar plates) were compared. DGGE of the resulting DNA harvest revealed highly variable 16S rRNA gene band patterns among the culture media tested (Fig. 4A). Two distinct clusters were observed at a similarity level of 0.62; one pooled all of the four tested plant-onlybased culture media, while the other contained the artificial culture media tested. Of particular interest is the DGGE pattern of surface-sterilized barley roots that clustered closer to those of the plant-only-based culture media tested, being distant from the artificial culture media. This is a strong indication of a higher degree of similarity in the *in situ* community structure of rhizobacteria within the barley root itself and those cultured on plant-only-based culture media (Fig. 4A). PCoA of Euclidean distances of related bacterial DGGE banding profiles showed the clear separation of three main groups (Fig. 4B). Along PCoA-1, reflecting a 41.03% variation, the two groups of plant-only-based culture media and plant roots clustered away from the third group of tested artificial culture media (nutrient agar, soil extract agar, and CCM). PCoA-2 reflected 25.04% variation and distinguished three separate groups of plant roots, plant-only-based culture media, and tested artificial culture media.

The number and pattern of DGGE bands of barley rhizobacteria differed according to the nature of culture media used. Based on the peak patterns, rhizobacteria diversity indices were computed. The values of the OTU richness (S′) as well as Shannon-Wiener index (H′) were positively related to the diversity of the rhizobacteria community, while the dominance index (D′) was negatively correlated (Table S2). The diversity indices reported higher values for the plantonly-based culture media (28–31 OTUs richness [S′] and 3.332–3.434 Shannon-Wiener indices [H′]), being closer to the values obtained for barley roots (27 OTUs richness [S′] and 3.296 Shannon-Wiener indices [H′]). The lowest values were obtained for the tested artificial culture media (17–22 OTUs richness [S′] and 2.833–3.091 Shannon-Wiener indices [H′]). Opposite results were reported for dominance values. Furthermore, a non-paired, heteroscedastic Student’s *t*-test showed highly significant levels (*P*=0.046) between the beta diversity distances of the tested artificial culture media vs. barley roots and plant-only-based culture media vs. barley roots. Deviations in all values of diversity indices (S′, H′, and D′) from surface-sterilized barley roots were calculated for all tested culture media, as shown in Fig. 4C. The tested plant only-based culture media generally reported the lowest standard deviation away from the barley roots, being the lowest for turfgrass powder culture media. In contrast, the highest values of deviations were scored for the tested artificial culture media.

Our results generally indicated the positive effects of the tested plant materials on the culturability and biomass production of rhizobacteria, particularly mowed turfgrass powder, which was reflected by the higher percentages of culturability and indices of diversity. This points to the idea that some groups of difficult-to-culture microbes have strong preferences for nutrients due to their streamlined genomes stripped of canonical metabolic pathways (15). This was further confirmed by the isolation of difficult-to-culture isolates of *Novosphingobium* sp., *Lysobacter* sp., and *Pedobacter* sp. among the rhizobacteria of Lucerne (*Medicago sativa* L.) when using plant medium based on homologous Lucerne plant materials (18). We suggest that the culturability of endo-rhizobacterial communities on plant-only-based culture media is linked to the type of host plant tested, supporting the use of a homologous culturing strategy. In the present study, turfgrass was genetically the closest species to the tested host plant, barley, which resulted in an increase in culturability and diversity (Fig. 4, Table S2). Further studies are needed in order to examine the extent to which plant-only-based culture media may be refined to efficiently accommodate or provide selectivity for the *in vitro* growth and biomass production of specific community compositions, particularly difficult-to-culture bacteria. A future strategy may be to avoid the use of heat stress treatments, *e.g.* drying and autoclaving, for the prepared culture media in order to restore their labile contents of nutrients and growth factors, *e.g.* amino acids and vitamins. This will contribute to the application of culturomic approaches to the study of the plant microbiome (25).

## Conclusion

The present study provided clear evidence for the potential use of more plant materials, in the form of refuse as cheap, available and sustainable bioresources, in the preparation of culture media rich in diverse stores of nutrients that will support the efficient *in vitro* culturing and biomass production of rhizobacteria. These plant materials have extended the challenge of plant-only-based culture media to be included in future culturomic studies on the plant microbiome. They are sufficient and efficient for culturing rhizobacteria for the purpose of escalating biomass production for future technologies of bio-preparations. They also satisfy crucial pre-conditions for laboratory and industrial use including cost-effectiveness and simplicity, matching what Leonardo da Vinci is reported to have said, “*Simplicity is the ultimate sophistication*” (https://www.brainyquote.com).

## Supplementary Material



## Figures and Tables

**Table 1:** Log numbers of CFU (data are log means±standard error [SE], *n*=3) of culturable rhizobacteria in different compartments of barley roots, compared to free soil, developed on various culture media; and bacterial quantification using qPCR of surface-sterilized barley roots. The mean value of qPCR cell numbers (obtained indirectly assuming that the average 16S rRNA gene copy number per cell is 3.6) is log 9.37±0.001 g^−^^1^ root dry weight obtained for four replicates.

Culture media	Endo-rhizosphere Log CFUs±SE (% of culturability)	Ecto-rhizosphere Log CFUs±SE	Free soil Log CFUs±SE
**Nutrient agar**	7.91±0.01^c^ (3.4)	8.19±0.11^d^	6.19±0.05^b^
**CCM**	7.96±0.09^c^ (3.9)	7.97±0.06^d^	6.16±0.03^b^
**Soil extract**	7.96±0.03^bc^ (3.8)	9.79±0.06^a^	6.20±0.02^b^
**Turfgrass powder**	8.59±0.09^a^ (16.3)	8.77±0.17^bc^	6.75±0.17^a^
**Clover powder**	7.95±0.08^c^ (3.8)	7.89±0.03^d^	6.24±0.16^b^
**Clover slurry**	7.89±0.02^c^ (3.3)	8.77±0.18^c^	5.48±0.18^c^
**Cactus powder**	8.16±0.08^b^ (6.1)	9.22±0.17^b^	6.00±0.03^b^
**Cactus slurry**	8.03±0.01^bc^ (4.5)	8.73±0.03^c^	6.08±0.05^b^
**LSD (*P*<0.05)**	0.1918	0.3797	0.3414

* Different letters indicate different significance levels.
