# Supplementary material for: Plant Materials are Sustainable Substrates Supporting New Technologies of Plant-Only-Based Culture Media for in vitro Culturing of the Plant Microbiota
Source: Microbes Environ. 2018 Mar 29;33(1):40–9. doi: 10.1264/jsme2.ME17135 (PMC5877342; doi:10.1264/jsme2.ME17135)
Supplement: Supplementary file 1 [file 33_40_s1.pdf]

**Table S1: The tested 16 rhizobacteria isolates**

| Original Strain code no. | Abbreviation used in the study | Species | Family | Source* |
|---|---|---|---|---|
| Azt B4 | A1 | *Azotobacter chroococcum* | *Pseudomonadaceae* | (2) |
| B43 | B1 | *Bacillus circulans* | *Bacillaceae* | (1) |
| 3B | B2 | *Bacillus circulans* | *Bacillaceae* | (2) |
| En 71/3 | B3 | *Bacillus licheniformis* | *Bacillaceae* | (3) |
| 21B | B4 | *Bacillus macerans* | *Bacillaceae* | (1) |
| 1E | B5 | *Bacillus polymyxa* | *Bacillaceae* | (2) |
| 30B | B6 | *Bacillus polymyxa* | *Bacillaceae* | (1) |
| NA 20 | B7 | *Bacillus subtilis* | *Bacillaceae* | (3) |
| K4 | E1 | *Enterobacter agglomerans* | *Enterobacteriaceae* | (2) |
| K3 | E2 | *Enterobacter agglomerans* | *Enterobacteriaceae* | (2) |
| 31Sh | K1 | *Klebsiella* sp. | *Enterobacteriaceae* | (1) |
| En 5/1 | P1 | *Pantoea* sp. | *Enterobacteriaceae* | (3) |
| Arbo 17 | Ps1 | *Pseudomonas aeruginosa* | *Pseudomonadaceae* | (3) |
| 34Sh | Ps2 | *Pseudomonas cepacia* | *Pseudomonadaceae* | (1) |
| Arbo 4 | Ps3 | *Pseudomonas fluorescens* | *Pseudomonadaceae* | (3) |
| Pseu. B6 | Ps4 | *Pseudomonas* sp. | *Pseudomonadaceae* | (2) |

## * For more information on the source of isolates and their characterization, please refer to the following references:


1. Othman, A.A., W.M. Amer, M. Fayez, and N.A. Hegazi. 2004. Rhizosheath of Sinai desert plants is a potential repository for associative diazotrophs. Microbiol. Res. 159:285–293.
2. Othman, A.A., W.M. Amer, M. Fayez, M. Monib, and N.A. Hegazi. 2003. Biodiversity of diazotrophs associated to the plant cover of north Sinai deserts. Arch. Agron. Soil Sci. 49:683–705.
3. Youssef, H.H., M.A. Hamza, M. Fayez, *et al.* 2016. Plant-based culture media: Efficiently support culturing rhizobacteria and correctly mirror their *in-situ* diversity. J. Adv. Res. 7:305–316.

**Table S2: OTUs Richness (S'), Shannon-Wiener index (H'), and Dominance (D') of rhizobacterial populations associated to barley roots and CFUs harvested on various culture media based on DGGE analysis.**

| Media | Richness Taxa (S') | Shannon (H') | Dominance (D') |
|---|---|---|---|
| **Barley root** | 27 | 3.296 ±0.159 | 0.037 ±0.011 |
| **Nutrient agar** | 22 | 3.091 ±0.168 | 0.045 ±0.015 |
| **CCM** | 18 | 2.890 ±0.177 | 0.056 ±0.018 |
| **Soil extract** | 17 | 2.833 ±0.178 | 0.059 ±0.020 |
| **Grass powder** | 28 | 3.332 ±0.156 | 0.036 ±0.011 |
| **Clover powder** | 30 | 3.401 ±0.153 | 0.033 ±0.010 |
| **Clover slurry** | 31 | 3.434 ±0.156 | 0.032 ±0.010 |
| **Cactus powder** | 31 | 3.434 ±0.153 | 0.032 ±0.010 |

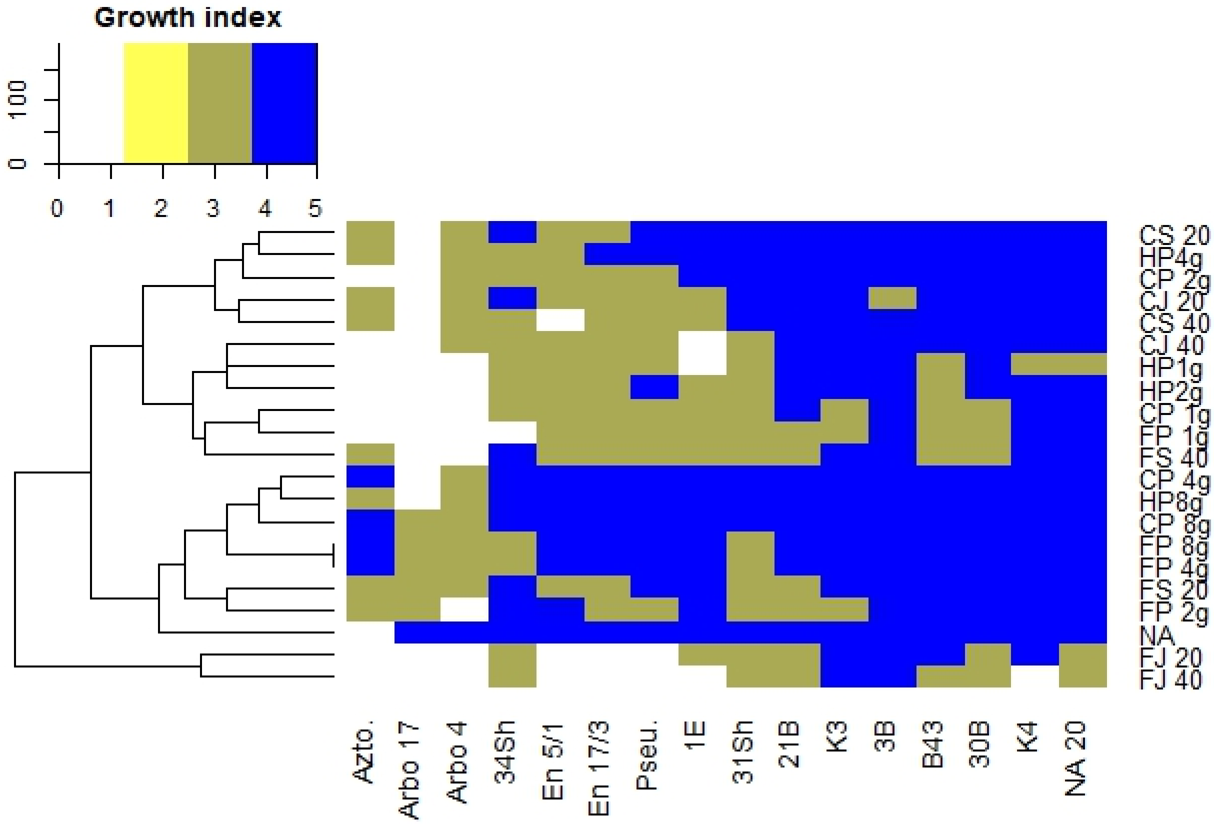


**Fig. S1:** Heatmap showing the growth indices [0, no growth; 1, scant (discontinued bacterial lawn, with scattered colonies); 2-3, good (continued bacterial lawn), and 4-5, very good (continued and very dense bacterial lawn)] of all tested isolates grown on 20 different formulations of plant-only culture media based on plant juices and slurries dilutions (1:20 or 1:40, v/v) as well as weights of dehydrated plant powders (1, 2, 4, or 8 g $L^{-1}$).

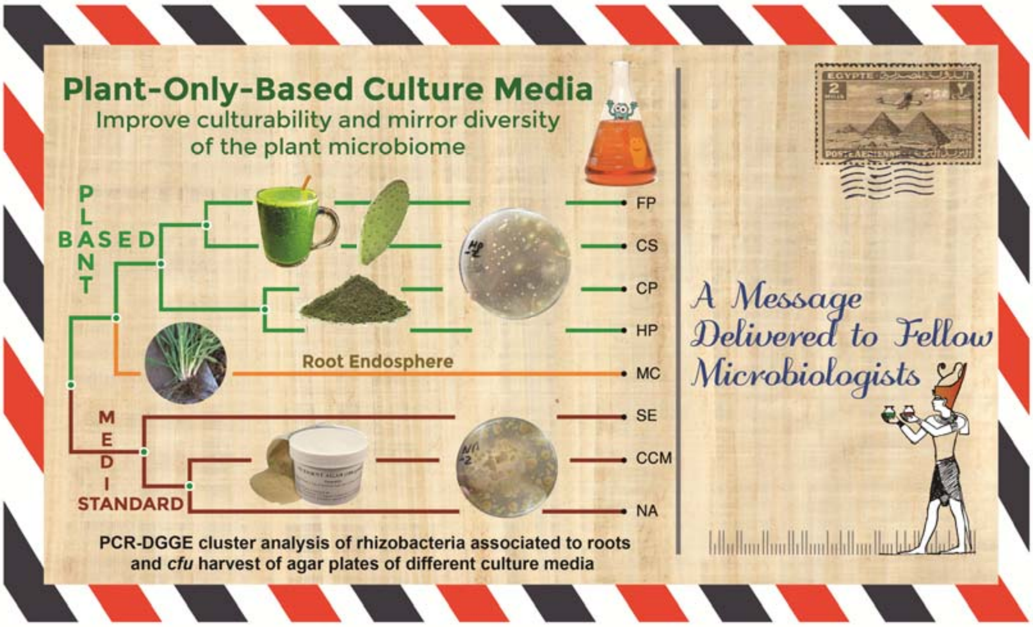


**Fig. S2:** Graphical abstract

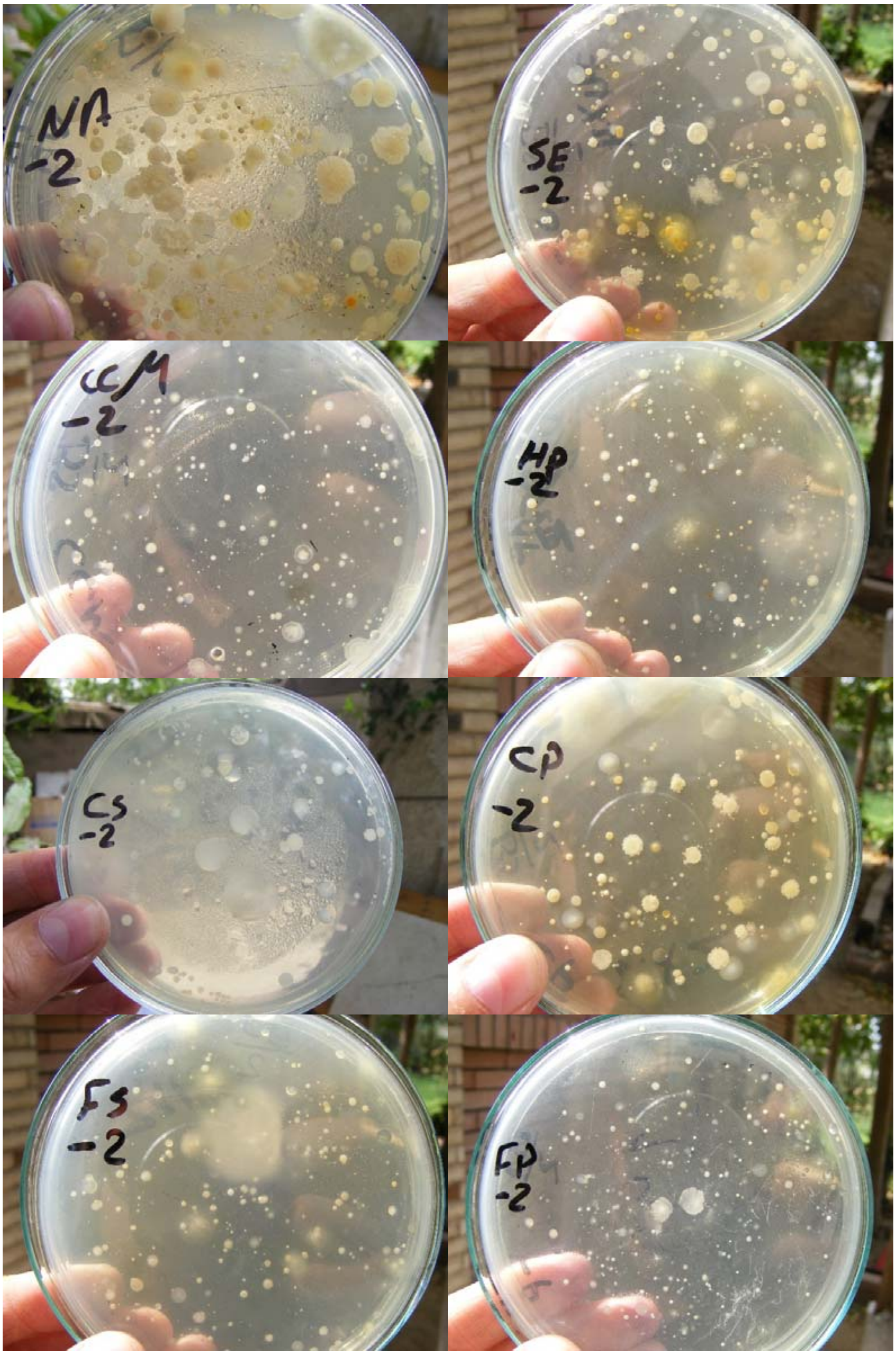


**Fig. S3:** CFU of endo-rhizobacteria as developed on the different tested culture media (comparing the same root dilution of $10^{-2}$): **NA**, nutrient agar; **SE**, soil extract agar; **CCM**, N-deficient combined carbon sources medium; **HP**, turfgrass powder, **CS**, clover slurry; **CP**, clover powder; **FS**, cactus slurry; **FP**, cactus powder.
